# Adequate Mentorship Program for Holmium Laser Enucleation of the Prostate (HoLEP) Leads to Satisfactory Short-Term Outcomes in the Early Learning Curve of Young Urologists: First-Year Outcomes of a Newly Established Mentorship Training in Mexico

**DOI:** 10.7759/cureus.41756

**Published:** 2023-07-12

**Authors:** Alan de Jesus Martinez-Salas, Oscar Uriel Garcia-Rivera, Irving Reyna-Blanco, Aldo Daniel Jimenez-Garcia, Hector Rosas-Hernandez

**Affiliations:** 1 Urology, Hospital Angeles Pedregal, Mexico City, MEX; 2 Urology, Centro Medico Nacional 20 de Noviembre Instituto de Seguridad y Servicios Sociales de los Trabajadores del Estado (ISSSTE), Mexico City, MEX; 3 Urology, Hospital General Instituto de Seguridad y Servicios Sociales de los Trabajadores del Estado (ISSSTE) Tlahuac "Dra. Matilde Petra Montoya Lafragua", Mexico City, MEX; 4 Urology, Corporativo Hospital Satélite, Mexico City, MEX

**Keywords:** holmium, lasers, prostatectomy, transurethral resection of prostate, prostatic hyperplasia, prostate

## Abstract

Introduction

Benign prostatic hyperplasia (BPH) is one of the most common causes of lower urinary tract symptoms in men. Holmium Laser Enucleation of the Prostate (HoLEP) has been recommended by international guidelines as an alternative to transurethral resection of the prostate (TURP). HoLEP’s learning curve and the lack of adequate mentorship remains an obstacle for the worldwide adoption of this technique.

Objective

To report the first-year learning curve of a newly established mentorship program in young urologists without any previous HoLEP experience.

Methods

We report a cohort of patients with BPH, with prostate size ≥70 grams, treated with HoLEP, analyzed for perioperative data and complications, and short-term postoperative complications and functional outcomes, at three and six months after surgery.

Results

A total of 47 patients were managed with HoLEP. Mean total operative time was 149.8 ± 42.9 minutes. We experienced five (10.6%) intraoperative complications, including one intravesical resection of the prostate with bipolar energy, three conversions to TURP and one conversion to open prostatectomy. We experienced four postoperative complications, all of them Clavien-Dindo ≤2. Median International Prostate Symptom Score (IPSS) decrease at six months was -17 points from baseline. Mean post-void residual volume and prostate-specific antigen significantly decreased by the third postoperative month. Multiple linear regression showed that prostate size is directly related to increased surgical time during the early learning curve.

Discussion

Our experience adequately reflects the importance of HoLEP mentorship in young urologists seeking training in this technique. Both surgeons had perioperative and postoperative outcomes deemed satisfactory based on previously published learning curves.

Conclusions

HoLEP is a technically difficult procedure, however, adequate mentorship leads to satisfying short-term outcomes since the early stages of the learning curve in young urologists with no previous training on this technique.

## Introduction

Benign prostatic hyperplasia (BPH) is one of the most common causes of lower urinary tract symptoms (LUTS) in men. In 2022, a systematic analysis published by the Global Burden of Disease Collaboration found a 70% increase in the overall prevalence of BPH cases in 2019 compared to 2000, rising from 55.1 million to 94 million cases in 2019 [1]. Most patients will be initially treated with medication, however, recent studies have concluded the clinical benefit and cost-effectiveness of initial surgical treatments for BPH [2-4]. When comparing multiple surgical treatments, holmium laser enucleation of the prostate (HoLEP) has proved to be the most cost-effective surgical option in the long term [5, 6].

Since the first experience published by Fraundorfer and Gilling in 1998, HoLEP became a milestone in endoscopic surgical treatments for BPH [7,8]. Initially, HoLEP was reserved for large-volume prostates. In time, several studies demonstrated that HoLEP is a perfect technique for BPH treatment regardless of prostate volume [9-11]. HoLEP has earned its place amongst the endoscopic BPH treatments, and current guidelines recommend HoLEP as an alternative to transurethral resection of prostate (TURP), for moderate-to-severe symptoms, regardless of prostate volume [12, 13].

Urologists’ experience with HoLEP continues to grow worldwide, however, in Mexico, access to this technique remains limited, mainly due to the absence of endoscopic enucleation of prostate (EEP) training during residency programs, and the lack of HoLEP experience by graduated urologists. Few hospitals in Mexico perform HoLEP, amongst which the Central Military Hospital has the highest volume in Mexico. They recently published their experience, with 62 HoLEP cases, in a one-year period, from 2018 to 2019, with satisfying outcomes [14].

Our hospital is a secondary care, general hospital of the public health system, it was inaugurated by the end of December 2020. On March 2021, the Urology Department was inaugurated, amidst the SARS-CoV-2 pandemic. In the first couple of months, due to the ongoing severity of the pandemic in Mexico, our hospital exclusively received COVID-19 patients, therefore Urology consultation and surgery started only in June 2021. HoLEP was introduced in our hospital for the first time in July 2021. The Urology Department comprises four young recently graduated urologists (less than two years from graduation). We decided to follow a cohort of patients managed with HoLEP since the beginning of our learning curve, to analyze the short-term outcome of patients treated with this technique in a newly established Urology Department of a general hospital, by a group of young urologists without any previous experience with HoLEP.

## Materials and methods

We retrospectively collected the data from a cohort of patients with LUTS secondary to BPH who were managed with endoscopic enucleation of the prostate (EEP) from July 2021 to August 2022. HoLEP was performed in all patients with prostate size ≥70 grams, with moderate to severe lower urinary tract symptoms, based on International Prostate Symptom Score (IPSS) ≥8 points, having already received at least three months of medical therapy (monotherapy or combined), and in all patients having already experienced recurrent acute urinary retention (AUR) with a previous unsuccessful removal of Foley catheter attempt. Based on our department protocols, patients exhibiting preoperative post-void residual volume (PVR) higher than 50% or 150 cm^3^ and all patients with a previous history of acute urinary retention are additionally studied with a renal ultrasound. If they exhibit bilateral renal pelvis dilation, they are sent to a tertiary care hospital for urodynamic analysis and management. Patients with prostate-specific antigen (PSA) greater than 4ng/mL or suspicious digital rectal exam are managed with transrectal ultrasound-guided biopsy before any endoscopic treatment. Demographic data, medical background, preoperative post-void residual volume (PVR), IPSS, prostate-specific antigen (PSA), and serum creatinine were recorded. We do not have uroflow or urodynamic studies in our hospital, therefore we were unable to perform uroflow analysis on our patients. Our primary outcome was postoperative short-term symptomatic evaluation, on the third and sixth months after surgery. IPSS was recorded in the first, third and sixth postoperative month. PSA and PVR were recorded in the third postoperative month. Our secondary outcomes included operative time, hospitalization time and perioperative and postoperative complications. Perioperative data, such as bleeding, enucleation time, morcellation time, and total operative time (the sum of enucleation + morcellation time) were recorded. Transoperative complications, and postoperative complications using the Clavien-Dindo classification, were recorded.

HoLEP technique and equipment

Upon the introduction of HoLEP in our Urology Department, we established a mentorship training program, with the assistance of an experienced urologist (HRH) from outside our hospital, with a background of more than 2000 procedures and over nine years of experience in HoLEP. All procedures were performed by two urologists from our team with no previous experience in HoLEP or any EEP technique, mentored by the invited surgeon (HRH). The first five procedures were performed with direct assistance from the mentor, stepwise, in a “mirror” fashion - one side of the prostate adenoma (usually the left side) was performed by the mentor, with step-by-step repetition of the right side of adenoma by the trainee urologist. Between the fifth and tenth procedures, the enucleation was entirely performed by the trainee with direct assistance from the mentor for each step if needed, to master each part of the technique satisfactorily. After the tenth procedure, the enucleation was completely performed by the trainee, the mentor was present during the surgery for step-by-step counseling, yet direct intervention from the mentor would only occur in case of technical difficulty encountered by the trainee, usually only reserved for unsatisfactory bleeding control using the laser.

We perform a modified version of the “En Bloc” technique described by Scoffone and Cracco in 2015, and later modified with an early apical release by Saitta et al. in 2019 [15, 16]. We begin the enucleation on the prostate apex, starting with a bilateral incision parallel to the verumontanum. We continue with bilateral apical lobar incision before the anterior apical incision described by Saitta et al., and then we proceed in a retrograde fashion enucleating all the adenoma bilaterally in an “En bloc” technique [16].

All surgeries were performed in an operating room with either general or spinal anesthesia.

The enucleation was carried out using a 60 Watt Versapulse Holmium:YAG laser system (Lumenis, Israel), a continuous flow 26Fr resectoscope with a laser fiber stabilizing sheath with a 30º optics scope (Karl Storz, Germany), and a 550nm fiber. Initial enucleation laser parameters were always the same, 1.2Hz with 40J, with occasional transoperative variation. Morcellation was performed with a 26Fr nephroscope with a 6º optics scope (Karl Storz), using a Hawk JAWS morcellator (Hawk, China). Bipolar endoscopic equipment (Karl Storz) was available on all procedures and was used by the end of each enucleation in case of unsatisfactory bleeding control with the laser, for bipolar cauterization.

Statistical analysis

Statistical analysis was performed with the SPSS version 29 (IBM Corp., Armonk, NY, USA). Variables were reported as means and standard deviation (SD) when continuous, or median and interquartile ranges (IQR) when ordinal with multiple categories (IPSS). Nominal variables were reported as frequencies and percentages. Student t-test was used for continuous variables. Friedman Test (ANOVA by Ranks) was used for ordinal variables with multiple categories. Median test with Yates correction was used for the comparative analysis of ordinal variables with multiple categories. Chi-square and Fisher's exact tests were used for nominal variables. Different multiple linear and logistic regression models were performed in search of the best significant prediction of the different postoperative outcomes. Two-sided p-values were used, with a significant level established at ≤0.05 value.

## Results

A total of 47 patients were treated with HoLEP from July 2021 to August 2022. The mean age was 72.5 ± 8.3 years. By the time of surgery, all patients had at least one medication for LUTS (alpha-blocker, 5-alpha-reductase inhibitors, or both) and 50% of the patients had an IPSS score of 20 or higher despite medication. Twenty-two patients (46.8%) had a previous history of AUR, and 20 patients (42.6%) had indwelling urinary catheter due to persistent AUR episodes. Eight (17%) patients had a previous history of macroscopic hematuria, and 10 (21.3%) patients had a previous history of urinary tract infection. Twenty-one (44.7%) patients had associated comorbidities. Two (4.3%) patients were on anticoagulation treatment. Only three patients (6.4%) had a previous history of surgical treatment for BPH, with transurethral resection of the prostate (TURP). Table 1 summarizes demographic and preoperative data.

**Table 1:** Demographic and preoperative data (n = 47 patients) Values expressed as mean ± standard deviation, median (interquartile range) or n (%). BMI: Body mass index; BPH: Benign prostatic hyperplasia; TURP: Trans-urethral resection of the prostate; AUR: Acute urinary retention; UTI: Urinary tract infection; IPSS: International prostate symptom score; PVR: Postvoid residual volume; PSA: Prostate-specific antigen.

Preoperative data	Value
Age	72.5 ± 8.3
BMI (kg/m^2^)	28.3 ± 2.4
Comorbidities	21 (44.7%)
Diabetes	6 (12.8%)
Hypertension	8 (17%)
Cardiac	3 (6.4%)
Other non-cardiac	4 (8.5%)
History of BPH surgery (TURP)	3 (6.4%)
History of AUR	22 (46.8%)
History of hematuria	8 (17%)
History of UTI	10 (21.3%)
Indwelling urinary catheter	20 (42.6%)
Anticoagulation	2 (4.3%)
Prostate weight (g)	106.1 ± 47.5
IPSS	20 (15-22)
Creatinine (mg/dL)	1.07 ± 0.53
PVR (mL)	76.4 ± 76.0
PSA (ng/dL)	5.62 ± 3.53

The first surgeon (AJMS) performed 18 procedures; the second surgeon (IRB) performed 29 procedures. There was no significant difference between both surgeons for enucleation time, total surgical time, intraoperative bleeding, or intraoperative and postoperative complications. Mean total operative time was 149.8 ± 42.9 minutes for all procedures, 156.7 ± 43.9 minutes for the first surgeon (AJMS) and 145.6 ± 42.4 for the second surgeon (IRB). We experienced five (10.6%) transoperative complications, including one morcellator dysfunction after complete enucleation, successfully managed with intravesical adenoma resection with bipolar equipment, three conversions to bipolar TURP secondary to surgical bleeding causing visibility problems, and one conversion to open prostatectomy secondary to morcellator dysfunction and bleeding. Postoperative complications occurred in four (8.5%) patients, three (60%) Clavien-Dindo 1, two postoperative lower urinary tract infections managed with outpatient antibiotics and one postoperative delirium, and one (40%) Clavien-Dindo 2, a postoperative bleeding successfully managed with transcatheter irrigation and blood transfusion. All patients were managed with a two-day hospital stay (48 hours following surgery) based on our pre-established protocols for TURP, due to our lack of previous experience with HoLEP. All patients were managed with a transcatheter continuous flow of 0.9% saline solution for 12-18 hours postoperatively, and the urinary catheter was removed 36 hours after surgery in all cases.

**Figure 1:**
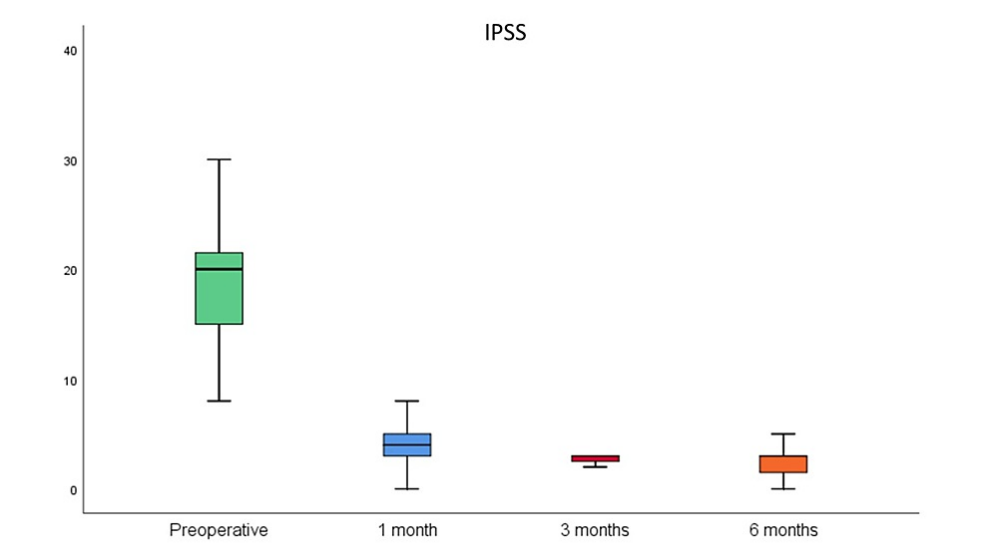
Evolution from preoperative IPSS to one, three, and six months of postoperative follow-up (p < 0.001). IPSS: International Prostate Symptom Score

Median IPSS after surgery was 4 points at the first month, and 3 at the third and sixth months, with a significant decrease from preoperative IPSS since the first postoperative month (-15 points) (p < 0.001), with an even better improvement at six months (-17 points) (p < 0.001) (Figure 1). Mean PVR at three months was 10.3 ± 8.8 milliliters (mL), with a significant improvement from preoperative PVR (p < 0.001), maximum postoperative PVR was 37mL. Mean PSA significantly decreased at three months (0.6 ± 0.3 ng/mL) (p < 0.001). The decrease in creatinine from baseline to three-month follow-up was not statistically significant (1.07 ± 0.53 mg/dL to 0.96 ± 0.19 mg/dL; p = 0.066). Seventeen (36.2%) patients referred postoperative dysuria after catheter removal, however by the first postoperative month this symptom had already disappeared or significantly improved in all patients. Thirty-five (74.5%) patients experienced postoperative urge urinary incontinence (UI), with a mean duration of 3.5 ± 3.3 months, the rate of urge UI at six months was 25.5% (12 patients). There were two cases of prostate cancer (4.3%), both Gleason Score 6, with preoperative PSA < 4.0ng/mL. Table 2 summarizes postoperative data.

**Table 2:** Perioperative and postoperative data (n=47 patients) Values expressed as mean ± standard deviation, median (interquartile range) or n (%). OP: Open prostatectomy; TURP: Trans-urethral resection of the prostate; PVR: Postvoid residual volume; IPSS: International prostate symptom score.

Perioperative data	Values			
	Total n=47	Surgeon 1 (AJMS) n=18 (38.3%)	Surgeon 2 (IRB) n=29 (61.7%)	P value
Operative time (min)	149.8 ± 42.9	156.7 ± 43.9	145.6 ± 42.4	0.396
Enucleation time	112.9 ± 35.0	116.7 ± 32.9	110.6 ±36.7	
Morcellation time	26.4 ± 17.3	30.78 ± 23.1	23.7 ± 12.2	
Transoperative complication	5 (10.6%)	1 (5.6%)	4 (13.8%)	0.636
Conversion to OP	1 (20%)		1 (25%)	
Conversion to TURP	3 (60%)		3 (75%)	
Intravesical resection of adenoma	1 (20%)	1 (100%)		
Bleeding (mL)	380.0 ± 187.4	400.6 ± 209.4	367.2 ± 175.0	0.559
Postoperative complications	4 (8.5%)	1 (5.6%)	3 (10.3%)	0.636
Clavien-Dindo 1	3 (60%)	1 (100%)	2 (66.7%)	
Clavien-Dindo 2	1 (40%)		1 (33.3%)	
Blood transfusion	1 (2.1%)		1 (3.4%)	
Dysuria	17 (36.2%)	5 (27.8)	12 (41.4%)	0.533
Incontinence	35 (74.5%)	11 (61.1%)	24 (82.8%)	0.168
IPSS 1 month	4 (3-5)	4 (2-4)	4 (3-7)	0.222
IPSS 3 months	3 (2-3)	3 (1-3)	3 (3-5)	0.613
IPSS 6 months	3 (1-3)	3 (1-3)	3 (3-4)	0.330
Creatinine (mg/dL)	0.96 ± 0.19	1.00 ± 0.07	0.89 ± 0.01	0.056
PSA (ng/dL)	0.61 ± 0.33	0.49 ± 0.07	0.67 ± 0.06	0.209
PVR (mL)	10.3 ± 8.8	12.3 ± 2.5	9 ± 1.3	0.065

A multiple linear regression model for predicting total surgical time, using prostate size, age, body mass index (BMI), and the number of procedures (R = 0.543; F = 4.393; p = 0.005; adjusted R square = 0.228), established a significant correlation between prostate weight and total surgical time (RC = 0.340; p = 0.014; 95% CI = 0.064-0.549). A multiple linear regression model for predicting the duration of postoperative incontinence using prostate size, age, BMI, surgeon (AJMS vs IRB) and the number of procedures (R = 0.613; F = 4.926; p < 0.001; adjusted R square = 0.299), established a significant negative correlation between the number of procedures and postoperative incontinence duration (RC = -0.370; p = 0.009; 95% CI = -0.271 - -0.041). Table 3 and Table 4 summarize both multiple regression models respectively. No logistic regression model was significant for the prediction of postoperative outcomes.

**Table 3:** Multiple linear regression model for the prediction of total operative time. RC: Regression coefficient; CI: Confidence interval for RC; BMI: Body mass index. *R = 0.543; F = 4.393; p = 0.005; adjusted R square = 0.228

Variable	RC	95% CI	P value
Age	0.252	-0.015 – 0.519	0.064
BMI (kg/m^2^)	-0.258	-0.526 – 0.011	0.59
Prostate weight (g)	0.340	0.071 – 0.608	0.014
Number of procedures	0.011	-0.257 – 0.279	0.933

**Table 4:** Multiple linear regression for the prediction of postoperative incontinence duration. RC: Regression coefficient; CI: Confidence interval for RC; BMI: Body mass index. *R = 0.613; F = 4.926; p < 0.001; adjusted R square = 0.299

Variable	RC	95% CI	P value
Age	-0.089	-0.348 – 0.170	0.493
BMI (kg/m^2^)	0.000	-0.257 – 0.256	0.998
Prostate weight (g)	-0.166	-0.438 – 0.107	0.226
Surgeon (AJMS vs IRB)	0.531	0.242 – 0.821	<0.001
Number of procedures	-0.370	-0.644 – -0.097	0.009

## Discussion

When Fraundorfer and Gilling published their initial experience with HoLEP, they reported a mean operative time of 98 minutes, ranging between 64 and 190 minutes, on different prostate volumes [7, 8]. Operative time in our cohort was higher than the early experience of the pioneers of this technique, however, mean surgical time during the learning curve of urologists without previous experience on HoLEP has previously been described to be as high as 138 minutes, on a learning curve of 100 consecutive cases [17]. Soto-Mesa et al. studied the learning curve of two urologists without any HoLEP mentoring and reported a mean operative time for the first 25 procedures of 234 minutes, reaching a plateau after 50 procedures, with a mean operative time of 132 minutes [18]. A multicenter study comparing the learning curve of nine different urologists reported a mean operative time of 106 minutes [19]. A recent paper published a seven-year experience of 31 different urologists, all of them mentored by two experienced surgeons, reporting a mean operative time of 80 minutes [20]. In our multivariate analysis, the only preoperative factor related to operative time was prostate size, as previously described by the literature [21, 22].

Transoperative complication rates during HoLEP learning curve, either conversion to transurethral resection of the prostate (TURP) and open prostatectomy (OP) or bladder and ureteric meatus lesions, may vary. In a systematic review performed by Kampantais et al., they reported rates of conversion to TURP as high as 30% on the first 30 procedures of the learning curve, decreasing to 10% after reaching 60 consecutive surgeries [21]. Another systematic review showed that conversion to TURP may be as high as 23% during the learning curve [23]. Recently, a high-volume center published a conversion rate to open prostatectomy of 2.4% [24]. We experienced five (10.6%) transoperative complications, all of them involving a certain type of conversion, either to TURP (four patients) or OP (one patient), similar to the rate of conversion experienced after a longer learning curve of 60 procedures, however, we had no bladder or ureteral meatus lesions [21].

Postoperative complication rates have been inversely associated to the learning curve, with varying rates according to the surgeon and publication, however, these may be as high as 40% during the first procedures [25]. A decrease in the complication rates may be achieved after completing 30 procedures, however, some authors establish this plateau to be as high as 80 consecutive surgeries [21, 25]. Two high-volume centers, one from Italy and one from France, published their experience on HoLEP, after achieving several years of expertise in this technique, reporting postoperative complication rates of 20% and 19%, respectively [20, 26]. Our complication rate was 8.5%. This may be attributable to our reduced number of patients, however, the adequate mentorship during our learning curve is an important factor leading to this low incidence of complications, Westhofen et al. already demonstrated a significantly lower incidence of complication rates when inexperienced urologists are mentored by an experienced surgeon compared to non-mentored trainees during their first 50 procedures (5% vs 16%) [17].

Postoperative urinary incontinence is a bothersome symptom commonly present after HoLEP. Experienced centers have published relatively low rates of incontinence, as low as 3.8% at 6-month follow-up, however, urinary incontinence is associated with the learning curve, with rates as high as 56% in inexperienced urologists [20, 21, 23, 26]. Our urinary incontinence rate was higher than the published literature, yet stress incontinence in our patients was negligible, referring to bothersome UI related to urinary urgency, with a mean duration of 3.5 months, inversely correlated with the number of procedures in the learning curve.

IPSS improvement was significant in our cohort, since the first month after surgery median IPSS decrease was 15 points, and 17 points at 6-month follow-up. Some meta-analyses have concluded the absence of statistical difference on functional outcomes between HoLEP and TURP for prostate sizes ≤100 grams, however, when analyzing the long-term efficacy of clinical functional outcomes of high-volume prostates, HoLEP is superior to TURP [9, 11, 27].

The retrospective nature of our study, as well as the low number of cases in comparison to high volume reference centers, are important limitations of our study, however, we believe that our experience adequately reflects the importance of HoLEP mentorship in young urologists seeking training in this technique, particularly in countries where HoLEP training is insufficient or not available during residency training. Even though none of the surgeons reached the threshold conventionally established to achieve an expertise on HoLEP (40 to 60 procedures), both surgeons had perioperative and postoperative outcomes deemed satisfactory based on previously published learning curves; this was particularly evident for the transoperative and postoperative complication rates and postoperative functional outcomes.

## Conclusions

We believe that HoLEP is bound to be the new “gold standard” for endoscopic treatment of BPH, the only remaining obstacle being the lack of EEP mentorship and training during some residency programs and for graduated young urologists, particularly in low and middle-income countries, therefore it should be an integral part of all urology residency training worldwide. As we demonstrated in our cohort, despite being a technically difficult procedure, adequate mentorship leads to satisfying short-term outcomes in HoLEP from the early stages of the learning curve, even for surgeons with no previous training on EEP.

We expect that our current experience will encourage young urologists without previous HoLEP experience to seek adequate mentorship to start their HoLEP learning curve.
